# Unilateral multifocal choroidal ischemia revealing a giant cell arteritis: a case report

**DOI:** 10.11604/pamj.2014.19.228.3132

**Published:** 2014-10-30

**Authors:** Zouheir Hafidi, Hanan Handor, Hamid Elmoussaif, Mina Laghmari, Abdelouahed Karmane, Samira Tachfouti, Rajae Daoudi

**Affiliations:** 1 Université Mohammed V Souissi, Service d'Ophtalmologie A de l'Hôpital des Spécialités, Centre Hospitalier Universitaire, Rabat, Maroc

**Keywords:** Giant cell arteritis, choroidal ischemia, multi focal, unilateral

## Brief

The authors report an unusual case of multifocal choroidal ischemia revealing a giant cell arteritis. A 70 year-old woman presented for a sudden blurry vision in her right eye. The medical anamnesis revealed a previous history of recurrent bilateral fugax amaurosis; she experienced jaw claudication and headache in the few past days, with febricula and generalized weakness.

**Figure 1:**
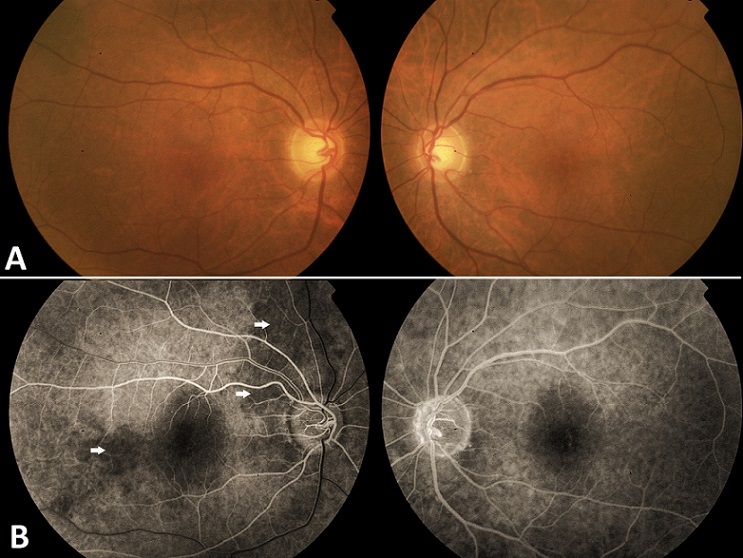
(A) funduscopic examination: there is no optic disc abnormalities, the rest of the retina appeared normal; (B) Fluorescein angiogram showing a blockage corresponding to a choroidal infarction in the right eye (white arrows)

Physical examination, revealed a visual acuity of 6/30 in the right eye and 6/6 in the left eye. Fundoscopy of both eyes was unremarkable, there was, particularly, no optic disk swelling (Figure 1, A). General examination showed a decreased temporal artery pulse on the right side. Fluorescein angiogram of the right eye showed blockage corresponding to a choroidal hypoperfusion (white arrows), and no abnormalities of the left eye (Figure 1, B).

The patient had a markedly increased erythrocyte sedimentation rate (ESR) (102 mm/hour), with a high level of C reactive protein (CRP) (45mg/dL). A biopsy of the right temporal artery was performed, and histological examination of the specimen showed a polymorphic inflammatory infiltrate associated with some multinucleated clusters of giant cells.

The patient was diagnosed with Giant cell arteritis, according to the aforementioned histological and clinical data, and received a prompt intravenous three days bolus of methylprednisolone at a dose of 1g per day, relayed by oral corticosteroids at 1 mg/kg/day, during 6 weeks (until normalization of the CRP and ESR) with gradual tapering. Over the next 6 months her visual acuity improved to 6/15 in the right eye.

### Temporal arteritis is a subacute systemic panarteritis with predilection over cranial arteries

Anterior ischemic optic neuropathy with or without the typical sectoral choroidal ischemia is the most common complication, leading to blindness unless it's not treated, with a high rate of bilateralization. Isolated choroidal ischemia without retinal vascular occlusion or acute anterior ischemic neuropathy is a rare manifestation of this disorder [1, 2], and is reported to be rather sectoral.

Unlike sectoral choroidal ischemia, which is, characteristically, due to the occlusion of the posterior ciliary arteries, multifocal choroidal ischemia is caused by the obstruction of the choriocapillaris [3]. Explaining the angiographic findings in this entity; mainly characterized by multifocal unsystematized hypo fluorescent areas, predominating around the optic disc, as noted in our case.

Numerous disorders may be associated to multifocal choroidal ischemia including toxemia of pregnancy [4], malignant hypertension [5], Vogt-Koyanagi-Harada disease [6], polyarteritis nodosa [7], and other systemic disorders [8, 9]. However it's not a common finding in giant cell arteritis, which affects large, medium, and small sized arteries, but not capillaries. Our documented case could reflect a possible involvement of choriocapillaris in giant cell arteritis.

Finally, the treatment should also be considered in such cases, because choroidal ischemia may precede optic nerve involvement [10].
